# Psychological Morbidity in Endometriosis: A Couple’s Study

**DOI:** 10.3390/ijerph182010598

**Published:** 2021-10-10

**Authors:** Maria Graça Pereira, Inês Ribeiro, Hélder Ferreira, Filipa Osório, Cristina Nogueira-Silva, Ana C. Almeida

**Affiliations:** 1 Psychology Research Center (CIPsi), School of Psychology, University of Minho, 4710-057 Braga, Portugal; ananevesalmeida@gmail.com; 2 School of Psychology, University of Minho, 4710-057 Braga, Portugal; inesnribeiro@gmail.com; 3 Department of Gynecology, Centro Materno-Infantil do Norte, Centro Hospitalar Universitário do Porto, 4099-001 Porto, Portugal; helferreira@hotmail.com; 4 Department of Gynecology and Obstetrics, Hospital da Luz, 1649-028 Lisboa, Portugal; filipabosorio@gmail.com; 5 Departament of Obstetrics, Gynecology and Reproduction Medicine, Hospital Santa Maria, 1649-028 Lisboa, Portugal; 6 Life and Health Sciences Research Institute, School of Medicine, University of Minho, 4710-057 Braga, Portugal; cristinasilva@med.uminho.pt; 7 ICVS/3B’s—PT Government Associate Laboratory, 4710-057 Braga, Portugal; 8 Department of Obstetrics and Gynecology, Hospital de Braga, 4710-243 Braga, Portugal

**Keywords:** endometriosis, psychological morbidity, patients, partners

## Abstract

Endometriosis is a chronic gynecological disease that impacts more than 176 million women worldwide, having a strong impact on psychological morbidity. This study aimed to evaluate the contribution of psychological morbidity, in women with endometriosis, taking into consideration the duration of the couple’s relationship and the duration of the disease and also examined whether women’s sexual satisfaction had an impact on their psychological morbidity (actor effect) and on their sexual partners’ psychological morbidity (partner effect) and vice versa. Participants were 105 women and their partners, who answered the Hospital Anxiety and Depression Scale (HADS); Couple Satisfaction Index (CSI-4) and the Global Measure of Sexual Satisfaction (GMSEX). The results revealed a direct effect between the perception of symptom severity, marital satisfaction, and women’s psychological morbidity. Sexual activity and the presence of infertility had an indirect effect on the relationship between sexual satisfaction, diagnosis duration, and psychological morbidity, respectively. Finally, women’s sexual satisfaction had a direct effect on their own and their partner’s marital satisfaction that predicted less psychological morbidity, in both. Thus, a multidisciplinary intervention focused on the couple’s sexual and marital relationship is needed to promote psychological well-being in this population.

## 1. Introduction

Endometriosis is a chronic and progressive inflammatory gynecological disease [1], often defined by the presence of endometrial tissue in locations outside the uterus, which can extend to any organ and induce chronic inflammatory reactions [2,3]. In the world, it is estimated to affect about 10% to 15% of the female population at reproductive age [4], which represents more than 176 million women [5,6]. In Portugal the incidence is estimated to be about 700,000 cases [7].

The main symptoms of endometriosis are menstrual irregularities, chronic pelvic pain, dysmenorrhea (cramps during the menstrual period), dyspareunia (pain during and after intercourse), and infertility [8]. However, symptoms have no equal expression in all women and about 3% to 22% of patients are asymptomatic [6]. Furthermore, symptoms may represent signs of other medical conditions, so the disease is usually diagnosed with a significant delay, on average seven years, which leads to irreversible functional and anatomical problems in women (e.g., reproductive organs) [9], with a major impact on their psychological well-being [10].

According to the American Society for Reproductive Medicine [11], endometriosis may be classified as minimal (stage I), light (stage II), moderate (stage III), or severe disease (stage IV), the latter being the most extensive stage of the disease. The classification is based on the size, depth, and location of the endometrial tissue implants, which reflect the extent of the disease [12]. There is, however, no association between the disease stage and the level of pain experienced by the patient [13].

The definitive diagnosis of endometriosis is made surgically, with the laparoscopic approach recommended for both diagnosis and treatment [13]. After surgical treatment, women reported a decrease in the intensity of dysmenorrhea, dyspareunia, and chronic pelvic pain after 6 and 12 months, with improvement in their emotional well-being [14,15].

Endometriosis is a physically and mentally incapacitating disease, with high costs on the patient, including adverse effects on intimate relationships, limitations in daily and social activities, and an increased risk of obstetric and neonatal complications [10,13]. On a psychological level, women develop anxious and depressive symptomatology (psychological morbidity). The relationship between endometriosis and anxiety/depression symptoms has been widely corroborated [16,17,18]. A systematic review of Barneveld et al. [19] showed that symptoms of depression and anxiety occur frequently in women with endometriosis, particularly related to chronic pain. Also, a recent study showed an increased risk of depressive disorders, anxiety, and stress [20] in women with endometriosis. Women suffering from dyspareunia and chronic pelvic pain reported high levels of anxiety and depression, showing the importance of considering the intensity of endometriosis symptoms when addressing the psychological impact of endometriosis [18,21]. The perceived severity of endometriosis has an impact on psychological morbidity. In a study by Martins et al. [22], perceived severity of symptoms played a moderating role in the relationship between chronic pelvic pain intensity and the patient’s quality of life. In addition, a negative association between psychological morbidity and quality of life was found [22].

Endometriosis impacts the patient’s partner as well [23,24]. According to Ameratunga et al. [23], 92% of partners reported negative emotions regarding the impact of the disease on their lives. In partners, the results of qualitative studies showed high depression and anxiety [24,25], as well as feelings of helplessness, frustration, worry and anger [25]. Partners of women with endometriosis reported their sexual life, intimacy and couple’s relationship being negatively affected by endometriosis, which may cause serious problems in their marital relationship. Relationship problems may become aggravated when there is a lack of communication about sexuality, sexual dysfunction as well as avoidance of sexual intercourse [26]. Endometriosis may also impact the couple’s relationship compromising the social support from male partners to women, which may seriously damage the relationship’s dyad [27]. However, literature is scarce in recognizing the impact of endometriosis in male partners as well and, as a result, there is also a lack of social support provided to partners [26].

In women with endometriosis, there is a decrease in the frequency of sexual intercourse, mainly due to dyspareunia, with a negative impact on marital satisfaction [28]. Moradi et al. [28] reported perceived lack of support from the partner in 50% of women that resulted in the ending of the marital relationship. Moreover, in asymptomatic women, infertility associated with endometriosis, or concerns about infertility, impacted the relationship, especially in young couples [29]. These results corroborate another study that revealed, in young couples, that 67% of women with endometriosis reported severe relationship problems and 19% ended their relationship due to disease symptoms [30].

There are no studies, to our knowledge, on the couple’s adaptation to endometriosis. In cancer studies, there is a positive association between women’s psychological morbidity and that of their partners [31,32]. The results of Ameratunga et al. [23] also reported the impact of endometriosis on the partner’s financial and sexual life, which ultimately had a major impact on marital satisfaction. An association between marital satisfaction and psychological morbidity, in the couple, has been reported in the literature, to the extent that the complications of endometriosis and its symptoms had an impact on the couple’s dynamics, at an emotional level [33].

Overall, recent studies emphasize the contribution of endometriosis to women’s sexual satisfaction, as the disease includes dyspareunia that compromises sexual activity [34]. Dyspareunia is associated with less frequent and difficulties in sexual intercourse, which contributes to decreased sexual satisfaction [34]. Tripoli et al. [35] found that 40% of women with endometriosis with dyspareunia experienced lower sexual satisfaction. Moreover, the presence of chronic pelvic pain was associated with an increase in sexual aversion and a decrease in the frequency of sexual intercourse, impacting sexual satisfaction. Martins et al. [22] also reinforced the contribution of frequency of sexual activity and marital satisfaction to sexual satisfaction.

Regarding partners, a study by Smith and Pukall [36], on women with dyspareunia, the results showed that partners were significantly less sexually satisfied compared to partners of women without dyspareunia. This result corroborates other studies showing a negative association between the frequency of sexual activity and partner’s sexual satisfaction [37,38]. However, De Graaff et al. [30], concluded that endometriosis did not have such an impact on a partner’s sexual satisfaction, since partners adjusted the frequency and intensity of sexual activity to their female partner’s limitations, compared to partners of women without the disease. Finally, a negative association was found between sexual satisfaction and anxiety/depression, in the couple [39].

Endometriosis seems to have an impact in women and their partners. However, studies to date have focused mainly on clinical symptoms, ruling out the influence of other variables in the couple, such as the perceived severity of symptoms, marital satisfaction, and sexual satisfaction. Nonetheless, it is important to assess how endometriosis impacts the woman, the partner, and the couple’s dyad, in order to promote psychological health in this population [28,40].

The present study was based on the Northouse et al.’s marital adjustment model [41] that includes personal, social, and illness-related factors, as antecedent factors of psychosocial adjustment to illness which are mediated by the cognitive appraisal of illness. In the present study, the sociodemographic variables such as the patient’s age and frequency of sexual activity were assessed. Social factors included the variables marital satisfaction and sexual satisfaction of both the patient and her partner. As a disease-related factor, the presence of infertility was considered. The perception of the severity of symptoms was included under the cognitive assessment of the disease and, finally, the adjustment to endometriosis included the psychological morbidity of the patient and the partner, as proposed by the model. As the impact of chronic pelvic pain on women’s quality of life is influenced by the duration of endometriosis [42] and also by the duration of the relationship with the partner, due to the difficulties that endometriosis imposes on the relationship [30], the present study assessed the moderating effect of such variables in the predictive model regarding psychological morbidity.

This study aimed to examine the indirect effect of frequency of sexual activity and the presence of infertility in the relationship between clinical variables (e.g., severity of symptoms; diagnosis duration) and marital/sexual satisfaction on women’s psychological morbidity taking into account the duration of the couple relationship and the duration of endometriosis; as well as the impact of women’s sexual satisfaction on their psychological morbidity (actor effect) and on their partners’ psychological morbidity (partner effect) and vice versa considering the indirect effect of marital satisfaction (Actor–Partner Interdependence Mediation Model—APIMeM.

The following hypotheses were raised: (1) the frequency of sexual activity and the presence of infertility as well as perception of symptom severity are expected to have an indirect effect between marital/sexual satisfaction and women’s psychological morbidity; (2) psychological morbidity will be higher in women with a shorter couple’s relationship and longer disease duration; (3) marital satisfaction is expected to have an indirect effect in the relationship between sexual satisfaction and psychological morbidity, and this effect is expected in the patient, partner and vice-versa.

## 2. Materials and Methods

### 2.1. Participants

The sample consisted of 105 women diagnosed with endometriosis, followed in the gynecology consultation of three main hospitals in Portugal and their partners. In 46.2% of women and 9.2% of partners, data were collected face-to-face. The study used a transversal design. Women’s sociodemographic and clinical characterization is shown in Table 1 and the partners’ sociodemographic characterization in Table 2.

### 2.2. Instruments

Sociodemographic and Clinical Questionnaires. For this study, two sociodemographic questionnaires were designed, one for women with endometriosis and another for their partners, assessing the sociodemographic variables (e.g., age, marital status, duration of the relationship, professional situation, frequency of sexual activity). Women also answered a clinical questionnaire that assessed clinical variables (e.g., perceived symptom severity, symptom intensity, stage of endometriosis).

Hospital Anxiety and Depression Scale (HADS; Zigmond and Snaith, [43]; Portuguese version of Sousa and Pereira, [44]) assesses anxiety and depression in clinical populations. The instrument includes 14 items divided into two subscales: HADS-D (even items), which measures depression symptoms, and HADS-A (odd items), which measures anxiety symptoms. The full scale provides an overall assessment that corresponds to psychological morbidity. Answers are rated in a 4-point Likert scale, where “0” corresponds to “not at all/never” and “3” corresponds to “very/always”, with a maximum score of 21 points for each subscale. For both subscales, scores between 0–7 are considered normal, between 8–10 mild, between 11–14 moderate, and between 15–21 severe. In the original version, Cronbach’s alpha was 0.76 for the anxiety subscale, 0.72 for the depression subscale and 0.89 for the full scale. The Portuguese version showed a Cronbach’s alpha of 0.87 for the full scale. In the present study, only the full scale was used with a Cronbach’s alpha of 0.91 for women and 0.86 for partners. 

Couples Satisfaction Index (CSI-4; Funk and Rogge, [45]; Portuguese version of Ferraz, Santos, Ribeiro and Pereira, [46]) assesses satisfaction with romantic/intimate partner(s). The instrument is a reduced version of the original version and is composed of four items. The first item is rated on a 7-point Likert scale, ranging from 0 to 6, and the remaining items are rated on a 6-point Likert scale, ranging from 0 to 5, with ratings ranging from 0 to 21 points. Higher scores indicate higher marital satisfaction. In addition, there is a cutoff point of 13.5, with equal or lower scores representing significant marital dissatisfaction. As for internal consistency, the original version found a Cronbach’s alpha of 0.92. The Portuguese version that included the sample of the present study found a Cronbach’s alpha of 0.81 for women and 0.69 for partners.

Global Measure of Sexual Satisfaction (GMSEX; Lawrance and Byers, [47]; Portuguese version of Pascoal et al. [48]) assesses sexual satisfaction through the subjective analysis that each participant makes of their current sexual relationship with a partner and consists of five items that rate the relationship according to a Likert scale of 7, with a maximum score of 35 (e.g., from a rating of “Very Good”—7 to 1—“Very Bad”). Higher scores indicate greater sexual satisfaction. The original version found a Cronbach’s alpha of 0.90 and the Portuguese version, with different samples (normative clinical online) found values between 0.83 and 0.94. In the present study, Cronbach’s alpha was 0.97 for patients and 0.96 for partners.

### 2.3. Procedure

This study was approved by the Ethics Committee for Research in Social Sciences and Humanities (CEICSH 013/2020) of the University of Minho and by the Ethics Committee of the hospitals where the data were collected.

Data was collected face-to-face in two hospitals, and online in another one. Women who fulfilled the criteria were invited to participate in the study by their gynecologist, who explained the purpose of the study and the procedures. In the hospital, where data collection took place online, the gynecologist asked the women’s email contact and participants were sent a link to answer the questionnaires online. The first online page presented the purpose of the study and the informed consent form, followed by the questionnaires. Participants had to sign the consent form to proceed to the sociodemographic questionnaire followed by HADS, CSI-4, and GMSEX. Women also gave their permission to include the partner in the study providing the partner’s email contact. The clinical questionnaire for all women was completed by their gynecologist during the consultation or after online collection. If the partner agreed to participate in the study, the online questionnaires would be sent, with a front page explaining the purpose of the study, followed by the informed consent form. The survey took about 10 min to complete.

Of the total number of women invited (*N* = 129), 12 refused to participate in the study due to time constraints. Regarding partners, 117 were invited, however, only 105 participated. Thus, the final sample consisted of 105 dyads (women with endometriosis and their respective partners).

### 2.4. Data Analysis

Taking into consideration the transversal design of the present study, the nomenclature indirect effect both in the path analysis and in the APIMeM was chosen regardless of the latter having the word mediation in the acronym.

The indirect effect of sexual activity frequency and infertility in the relationship between clinical (e.g., intensity of dysmenorrhea), psychological variables and the dependent variable was analyzed through a path analysis, taking into consideration the Northouse model [41]. The adequacy of the theoretical model was examined through the follow fit indexes: χ^2^, Goodness Fit Index (GFI), Tucker-Lewis Index (TLI), Comparative Fit Index (CFI), Root Mean Square Error of Approximation (RMSEA) and Standardized Root Mean Square Residual (SRMR) indexes [49]. Adequate fit indexes for χ^2^ should be non-significant, for the GFI, TLI and CFI above 0.95, and for the RMSEA and SRMR below 0.60 and 0.80, respectively [49].

To test the moderating effect of the duration of endometriosis and the duration of the relationship with the sexual partner, a multigroup analysis was performed using: less than 40 months regarding the duration of endometriosis (*n* = 54) versus more than 40 months (*n* = 51) and less than 11 years regarding the duration of the relationship (*n* = 58) versus more than 11 years (*n* = 47), respectively (based on the mean for both variables). The results of the adjusted model without any constrains and the fully constrained model need to be significantly different to prove the moderating effect.

To analyze the indirect effect of the marital satisfaction, the version of APIMeM [50] with distinguishable dyads [51] was used. The APIMeM model included six variables and examined the indirect effect of marital satisfaction of women with endometriosis and their partners (two mediating variables) on the relationship between women’s and partners’ sexual satisfaction (two independent variables) and women’s and partners’ psychological morbidity (two dependent variables). This model was performed without standardization of the scales and the regression values were used to analyze data. To examine the fit of the APIMeM model to the data, the ratio χ^2^*/DF,* should be less than 2, the CFI and the RMSEA should be the same as those described above for the path analysis model [52]. The nonsignificant paths were removed, allowing the estimation of the final model.

To analyze the indirect effect of marital satisfaction in the APIMeM, the bootstrap technique with 3000 samples and 95% of confidential intervals (95% CI) was performed (the interval should not include zero to be significant [53]).

The path analysis, multigroup analysis, and APIMeM analysis were performed with the Amos version 22 of SPSS, using maximum likelihood estimation.

## 3. Results

### 3.1. The Indirect Effect of Infertility and Frequency of Sexual Activity on Women’s Psychological Morbidity—Path Analysis

**Figure 1:**
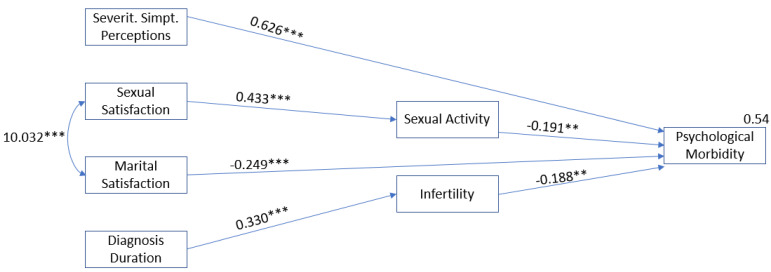
Path Analysis (final model) in women with endometriosis (*n* = 105). Note. Only the significant paths are shown in the figure. The final model showed good fit to the data: χ^2^(14) = 18.379; *p* = 0.190; GFI = 0.956; TLI = 0.952; CFI = 0.968; RMSEA = 0.055 (0.000; 0.116); *p* = 408. ** *p* < 0.01; *** *p* < 0.001.

The results showed that the frequency of sexual activity had an indirect effect on the relationship between women sexual satisfaction and psychological morbidity of women with endometriosis (β = −0.083; *p* = 0.002). Moreover, infertility has an indirect effect in the relationship between diagnosis duration and women’s psychological morbidity (β = −0.062; *p* = 0.009). The final model also showed a direct effect between perception of severity of symptoms (β = 0.626; *p* = 0.001) and marital satisfaction (β = −0.249; *p* = 0.001) with psychological morbidity of women with endometriosis (Table 3, Figure 1).

The final model revealed good fit indexes: χ^2^(14) = 18.379; *p* = 0.190; GFI = 0.956; TLI = 0.952; CFI = 0.968; RMSEA = 0.055 (0.000; 0.116); *p* = 408, explaining 54% of the variance of psychological morbidity. 

### 3.2. Multigoup Analysis

The results of the adjusted model without any constrain and the model with full constrains showed that the models were not significantly different, suggesting that either the duration of endometriosis (Δχ^2^ (6) = 1.841; *p* > 0.05) or the duration of the relationship with the sexual partner (Δχ^2^ (6) = 4.637; *p* > 0.05) had no moderating effect, in both analyses.

### 3.3. Direct and Indirect Effects in the Actor–Partner Interdependence Mediation Model (APIMeM)

The initial model did not fit well the data, (χ^2^(6) = 127.263; *p* = 0.000; χ^2^*/DF* = 8.484; CFI = 1.000; RMSEA = 0.268 (0.266; 0.312); *p* = 408). The preliminary analysis (see Table 4) regarding the actor effects showed women’s sexual satisfaction positively correlated with their marital satisfaction and negatively with psychological morbidity. In partners, sexual satisfaction correlated positively with marital satisfaction and negatively with psychological morbidity. Regarding partner effects, partner’s sexual satisfaction correlated positively with women’s marital satisfaction and negatively with women’s psychological morbidity.

**Figure 2:**
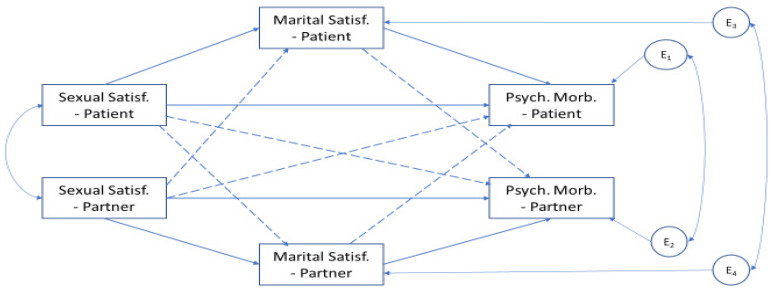
The conceptual actor–partner interdependence model with mediation in the relationship between sexual satisfaction, marital satisfaction, and psychological morbidity of women and their sexual partners (*n* = 105 dyads). Note: the solid arrows represent the actor effect and the dashed arrows represent the partner effect.

**Figure 3:**
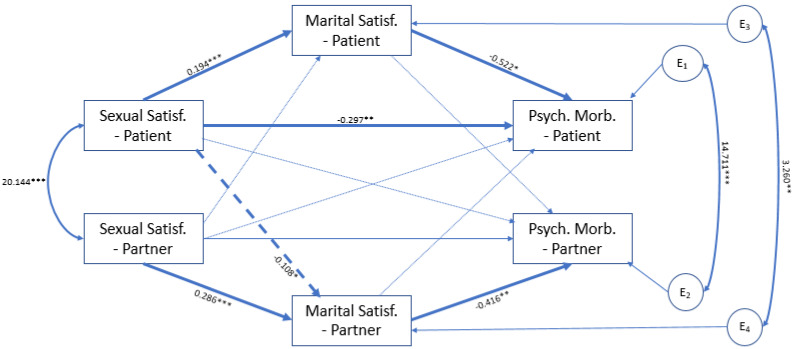
Final Model of APIMeM. Note. Significant relationships between sexual satisfaction, marital satisfaction and psychological morbidity in women and their partners are represented by dark arrows. Solid arrows indicate the actor effect and dashed arrows indicate the partner effect. Light colored arrows indicate the nonsignificant paths. * *p* < 0.05; ** *p* < 0.01; *** *p* < 0.001.

Figure 2 depicts the model initially tested. After the trimmed of the nonsignificant paths in the model, represented in light color arrows (Figure 3), the final model showed a good fit to the data (χ^2^(8) = 8.918; *p* = 0.349; χ^2^*/DF* = 1.115; CFI = 0.992; RMSEA = 0.033 (0.000; 0.123); *p* = 0.533, and explained 16% of the women’s psychological morbidity and 6.5% of the sexual partner’ psychological morbidity.

Regarding the actor effect, in women, a direct effect between women’ sexual satisfaction and psychological morbidity was found. An indirect effect between sexual satisfaction and psychological morbidity through marital satisfaction in both women and partners was also found. Regarding the partner effect, only a significant direct path between women’s sexual satisfaction and the partner’s marital satisfaction was found.

According to the results, women’s sexual satisfaction had an effect on both women and the sexual partner’s psychological morbidity through women’s marital satisfaction (β = −0.105; SE = 0.0035; 95% CI = −0.186 to −0.043; *p* = 0.001) and partner’s marital satisfaction (β = 0.033; SE = 0.020; 95% CI = 0.003 to 0.084; *p* = 0.028), respectively. In turn, partner’s sexual satisfaction had an impact on their own psychological morbidity through their marital satisfaction (β = −0.119; SE = 0.048; 95% CI = −0.229 to −0.038; *p* = 0.005).

## 4. Discussion

In women with endometriosis, the results showed an indirect effect of the frequency of sexual activity in the relationship between sexual satisfaction and psychological morbidity. Indeed, sexual activity in women with endometriosis who have dyspareunia is reduced, and women experience less sexual satisfaction [34]. In addition, the frequency of sexual activity, the presence of pain during sexual interaction and sexual dissatisfaction may create some apprehension in the patient, being associated with the development of anxiety and depression symptoms [34,39]. Therefore, it is important to ask women about their sexual life in routine clinical assessments.

Results also showed an indirect effect of infertility in the relationship between the duration of endometriosis and women’s psychological morbidity. Concerning infertility, in endometriosis, anxious and depressive symptoms are associated with an increase in the ineffectiveness of clinical treatments to resolve infertility [13,54]. In this sense, the longer the duration of the disease without an efficient treatment or infertility resolution may be associated with increased psychological morbidity. The discussion of infertility and monitorization of anxiety and depressive symptoms is paramount regarding the intervention in women with endometriosis.

The final path analysis model showed a direct effect between perceived severity of symptoms and marital satisfaction with psychological morbidity in women with endometriosis. Studies confirm that a perception of endometriosis symptoms as severe can exacerbate anxiety and depression symptoms [55,56]. In addition, many women with endometriosis report reduced social support and more relational difficulties with their partners, showing less marital satisfaction [28,30], which in turn is associated with increased anxious and depressive symptoms [33]. Therefore, it is important to assess and help women to increase their social support in order to help decrease psychological morbidity.

Contrary to our expectations, there was no moderating effect of the duration of endometriosis diagnosis or the duration of the relationship with the sexual partner. The literature shows that a delay in the corrects endometriosis diagnosis, that may last in average seven years [9] leads to several problems on a physical and psychological level and is, therefore, associated with negative emotions [10], but after diagnosis the duration of the disease may no longer have a significant expression, as the disease and treatment options become known. Regarding the duration of the relationship with the partner, other variables such as marital satisfaction and sexual satisfaction may have a more significant effect and, therefore, explain the presence of psychological morbidity [33,34] regardless of the duration of the relationship with the partner. More studies with a longitudinal design are needed to understand the influence of the duration of the couple’s relationship [24], as well as the impact of the duration of endometriosis, over time.

Regarding the dyadic analysis, the final model showed that the woman’s sexual satisfaction had an effect on her psychological morbidity through marital satisfaction and on the partner’s psychological morbidity through the partner’s marital satisfaction. Low frequency of sexual activity and sexual dissatisfaction in patients with dyspareunia, due to the presence of pain and difficulties during sexual intercourse, have been associated with higher psychological morbidity in women [18,33]. In addition, women with less marital satisfaction may experience greater difficulties during intercourse, which are associated with greater anxious and depressive symptoms [29,33]. The literature emphasizes the importance of sexuality, both individually and in the marital relationship [18], therefore, it makes an intuitive sense that a patient’s sexual satisfaction may have an influence on a partner’s sexual satisfaction and indirectly (through marital satisfaction) on psychological morbidly. Interestingly, this was only true for women and not for partners. However, contrary to our expectations, partner sexual satisfaction had no effect on psychological morbidity in women. Perhaps other variables such as disease symptoms [19] and sexual satisfaction [57] may have a greater impact on women’s psychological morbidity, overriding the effect of partner sexual satisfaction. More studies are needed to confirm this hypothesis.

Partners’ sexual satisfaction had an impact on their psychological morbidity through the partner’s marital satisfaction. This result is in accordance with the literature, as lower marital satisfaction in partners has been associated with lower sexual satisfaction and higher psychological morbidity, particularly in partners of women who have dyspareunia or chronic pelvic pain [38,39]. Regarding the partner effect, the results also showed a significant direct path between women’s sexual satisfaction and the partner’s marital satisfaction. Ameratunga et al. [23] reported that partners are significantly affected in their sexual life with a great impact on marital satisfaction. In another study, women’s sexual satisfaction was associated with less difficulty in sexual activity [34] and partner’s marital satisfaction, in couples coping with provoked vestibulodynia [38]. Patient and partner’s psychological morbidity were also positively correlated, and this result is consistent with the literature, showing that the couple experienced a spectrum of emotions together such as anxiety and depression [24], helplessness, worry and anger [25], which may also negatively affect their sexual function and intimacy, compromising the couple’s relationship [26].

Women’s marital satisfaction had no indirect effect in the relationship between partners’ sexual satisfaction and their psychological morbidity. Despite the difficulties presented by women with endometriosis, partners may be able to adapt to disease limitations regarding sexual activity [57] with no repercussions on psychological morbidity. More studies are needed to assess the relationships between these variables, over time.

Finally, as expected, the results showed a direct effect between women’s sexual satisfaction and their psychological morbidity. This result is supported by the literature that shows a strong association between sexual satisfaction and psychological morbidity in women with endometriosis [24]. In patients with dyspareunia, this association is even more significant due to the presence of pain during sexual activity and the inability to have sexual intercourse that direct impacts psychological well-being, exacerbating anxious and depressive symptoms [18,21]. Therefore, it is important that intervention in endometriosis addresses ways to improve sexual satisfaction in order to decrease psychological morbidity.

This study presents limitations that need to be acknowledged. First, the cross-sectional design and the small sample size, which does not allow causal inference and the generalization of the results, respectively. In addition, only self-report instruments were used. Future studies should focus on the impact of the endometriosis diagnosis and treatment considering both the female and male’s perspectives, addressing the influence of the disease on sexual function, intimacy and relationship dynamics. Despite the limitations, the study contributes to the understanding of the impact of endometriosis in women and their partners not only at a theoretical level, but also for intervention.

## 5. Conclusions

According to the results, the intervention should focus on clinical variables, such as perceived symptom severity, marital and sexual satisfaction in order to reduce psychological morbidity in women with endometriosis particularly in those with a lower frequency of sexual activity and infertility due to the disease. Routine gynecological consultations should address and monitor women’s concerns regarding infertility and sexual activity, since these variables are important for women’s psychological morbidity. Male partners’ perceptions and sexual function should also be addressed, as women’s endometriosis may impact profoundly the couple’s intimacy and dynamics requiring social support to adapt to the disease and its treatment.

The results also showed the importance of women’s sexual satisfaction on her psychological morbidity through marital satisfaction and on the partner’s psychological morbidity through the partner’s marital satisfaction, suggesting the importance of marital satisfaction in endometriosis. In fact, partner’s psychological morbidity is influenced by the women’s sexual satisfaction while the other way around was not found. Therefore, the results emphasize the importance of assessing women with endometriosis together with their partners, in a dyadic context. Treatment for endometriosis should address sexual satisfaction and marital satisfaction in order to decrease psychological morbidity in patients and partners and promote a better adjustment to the disease. For those couples struggling with adaptation to endometriosis, at the marital/sexual levels, couple’s therapy should be recommended.

## Figures and Tables

**Table 1:** Sociodemographic and clinical characterization of the women.

Sociodemographic Characterization	*N* (%)	Mean (SD)	Minimum	Maximum
Age	105	36.08 (6.11)	23	51
Years of Education	105	14.52 (3.46)	6	21
Frequency of sexual activity/month	105	6.63 (5.59)	0	30
Duration of the relationship	105	11.26 (6.65)	1	29
**Nationality**				
Portuguese	103 (98.1)			
Brazilian	2 (1.9)			
**Marital Status**				
Married/Cohabiting	88 (83.8)			
Single	11 (10.5)			
Divorced	6 (5.7)			
**Professional Status**				
Actively employed	61 (93.8)			
Unemployed	3 (4.6)			
Retired	1 (1.5)			
Absence from work	78 (74.3)			
**Clinical Characterization**				
Dysmenorrhea intensity	105	5.10 (4.11)	0	10
Dysuria intensity	105	0.85 (2.34)	0	10
Dyschezia intensity	105	2.51 (3.67)	0	10
Dyspareunia intensity	105	3.90 (3.84)	0	10
Chronic pelvic pain intensity	105	2.81 (3.37)	0	10
Duration of diagnosis (months)	105	60.00(60.53)	2	360
Having Surgery	75 (71.4)	33.37 (37.66)		
Infertility	37.1(37.1)			
**Perception of Symptom Severity**				
Light	19 (18.4)			
Moderate	38 (36.9)			
Severe	46 (44.7)			
**Stage of disease**				
I	1 (1.0)			
II	13 (12.4)			
III	29 (27.6)			
IV	62 (59.0)			

**Table 2:** Sociodemographic characterization of the partners.

Sociodemographic Characterization	*N* (%)	Mean (SD)	Minimum	Maximum
Age	105	37.73 (7.43)	20	59
Years of Education	105	12.81 (3.62)	4	22
**Nationality**				
Portuguese	103 (98.1)			
Brazilian	2 (1.9)			
**Professional Status**				
Actively employed	101 (96.2)			
Unemployed	4 (3.8)			

**Table 3:** Indirect effects of sexual activity and infertility in women with endometriosis.

Predictor	Indirect Effect	Outcome	β	SE	95% CI		*p*
					LL	UL	
Sexual Satisfaction	Sexual Activity	Psychological Morbidity	−0.083	0.028	−0.142	−0.029	0.002
Diagnosis Duration	Infertility	Psychological Morbidity	−0.062	0.032	−0.139	−0.015	0.009

Note: SE—standard error; 95% CI—95% of confidence interval; LL—lower limit; UL—upper limit.

**Table 4:** Correlations, means, and standard deviations of the variables in the model.

	1	2	3	4	5	6
1. Sexual Satisfaction—Women	---					
2. Sexual Satisfaction—Sexual Partner	0.431 ***	---				
3. Marital Satisfaction—Women	0.390 ***	0.262 **	---			
4. Marital Satisfaction—Sexual Partner	0.007	0.453 ***	0.306 **	---		
5. Psychological Morbidity—Women	−0.365 ***	−0.270 **	−0.357 ***	-023	---	
6. Psychological Morbidity—Sexual Partner	−0.072	−0.233 *	−0.187	−0.242 *	0.354 ***	---
Mean	27.83	27.41	15.76	15.44	15.04	10.38
SD	7.23	6.53	3.59	3.61	8.42	5.77

*p* < 0.05; ** *p* < 0.01; *** *p* < 0.001.

## Data Availability

Data available on request due to restrictions, e.g., privacy or ethical. The data presented in this study are available on request from the corresponding author.
